# Birth order and prosociality in the early adolescent brain

**DOI:** 10.1038/s41598-021-01146-0

**Published:** 2021-11-08

**Authors:** Naohiro Okada, Yu Yamamoto, Noriaki Yahata, Susumu Morita, Daisuke Koshiyama, Kentaro Morita, Kingo Sawada, Sho Kanata, Shinya Fujikawa, Noriko Sugimoto, Rie Toriyama, Mio Masaoka, Shinsuke Koike, Tsuyoshi Araki, Yukiko Kano, Kaori Endo, Syudo Yamasaki, Shuntaro Ando, Atsushi Nishida, Mariko Hiraiwa-Hasegawa, Charles Yokoyama, Kiyoto Kasai

**Affiliations:** 1 Department of Neuropsychiatry, Graduate School of Medicine, The University of Tokyo, Hongo 7-3-1, Bunkyo-ku, Tokyo, 113-8655 Japan; 2 International Research Center for Neurointelligence (WPI-IRCN), The University of Tokyo Institutes for Advanced Study (UTIAS), The University of Tokyo, Hongo 7-3-1, Bunkyo-ku, Tokyo, 113-0033 Japan; 3 Department of Psychiatry and Behavioral Sciences, Tokyo Metropolitan Institute of Medical Science, Kamikitazawa 2-1-6, Setagaya-ku, Tokyo, 156-8506 Japan; 4 Institute for Quantum Life Science, National Institutes for Quantum and Radiological Science and Technology, Anagawa 4-9-1, Chiba, 263-8555 Japan; 5 Department of Molecular Imaging and Theranostics, National Institute of Radiological Sciences, National Institutes for Quantum and Radiological Science and Technology, Anagawa 4-9-1, Chiba, 263-8555 Japan; 6 Office for Mental Health Support, Mental Health Unit, Division for Practice Research, Center for Research On Counseling and Support Services, The University of Tokyo, Hongo 7-3-1, Bunkyo-ku, Tokyo, 113-8655 Japan; 7 Department of Psychiatry, Teikyo University School of Medicine, Kaga 2-11-1, Itabashi-Ku, Tokyo, 173-8606 Japan; 8 The University of Tokyo Institute for Diversity and Adaptation of Human Mind (UTIDAHM), The University of Tokyo, Komaba 3-8-1, Meguro-ku, Tokyo, 153-8902 Japan; 9 Department of Child Psychiatry, Graduate School of Medicine, The University of Tokyo, Hongo 7-3-1, Bunkyo-ku, Tokyo, 113-8655 Japan; 10 Department of Evolutionary Studies of Biosystems, School of Advanced Sciences, The Graduate University for Advanced Studies (SOKENDAI), Shonan Village, Hayama, Kanagawa 240-0193 Japan

**Keywords:** Neural circuits, Social behaviour

## Abstract

Birth order is a crucial environmental factor for child development. For example, later-born children are relatively unlikely to feel secure due to sibling competition or diluted parental resources. The positive effect of being earlier-born on cognitive intelligence is well-established. However, whether birth order is linked to social behavior remains controversial, and the neural correlates of birth order effects in adolescence when social cognition develops remain unknown. Here, we explored the birth order effect on prosociality using a large-scale population-based adolescent cohort. Next, since the amygdala is a key region for sociality and environmental stress, we examined amygdala substrates of the association between birth order and prosociality using a subset neuroimaging cohort. We found enhanced prosociality in later-born adolescents (N = 3160), and observed the mediating role of larger amygdala volume (N = 208) and amygdala-prefrontal functional connectivity with sex-selective effects (N = 183). We found that birth order, a non-genetic environmental factor, affects adolescent social development via different neural substrates. Our findings may indicate the later-born people’s adaptive survival strategy in stressful environments.

## Introduction

In family psychological studies, the positive effect of being earlier-born on cognitive intelligence was established^1–3^. This phenomenon can be accounted for by the resource dilution model positing that parental resources are limited, thus the resource allocation to each child declines as sibling number and birth order increase^4^. A historical debate has centered on a potential birth order effect in social ability, where later-born siblings have putative higher levels of social development^5^. In addition, the recent report on reduced problem behaviors in later-born children suggested that they may be advantaged in developing positive social behavior through interactions with older siblings^6^. In contrast, later-born children are less likely to feel secure in a family environment compared to first-born adolescents, due to sibling competition^7,8^ or reduced time spent by mothers in affectionate attachment with their later-born children^9^.

Prosocial behavior (PB) or prosociality, defined as “voluntary behavior intended to benefit another”^10^, is considered a measurable benchmark of healthy social development. PB is a fundamental basis for human social interaction. PB can be accounted for by the cost benefit model such as indirect reciprocity^11^ and the group selection theory such as the strong reciprocity model^12^. PB is a factor related to psychological resilience^13^. PB strengths protect against depressive symptoms during the adolescent period^14^. This is at least partly because PB is associated with having someone to talk to if there was a problem^15^. Although many studies have documented the importance of parent–child interaction for the promotion of PB^16^, children’s source of prosocial learning is not limited to parents, and may extend to competent others generally^17^. Older siblings, who are usually more competent than their younger siblings, may also be a source of prosocial learning in family environment, but a definitive resolution of the potential association between birth order and prosociality remains controversial with divergent results attributed to differences in target generation^18,19^. Previous studies revealed that PB increases rather than decreases under stress, through which it may be hypothesized that later-born people, who are less likely to feel secure, are relatively prosocial^20,21^. Prosociality emerges in childhood, develops until early adolescence, slightly declines during middle adolescence, and bounces during late adolescence^22,23^. This suggests that focusing on this critical window of pre-teenage and early teenage development would be helpful in resolving the effect of birth order on prosociality.

Only recently have the neuroanatomical and neurofunctional correlates of prosociality in adolescence been reported using magnetic resonance imaging (MRI). Previous adolescent structural MRI (sMRI) studies demonstrated that prosociality is associated with a thicker cortex in the lateral orbitofrontal cortex/pars orbitalis, pre-/postcentral cortex^24^, superior frontal cortex and middle frontal cortex^25^. In addition, higher prosociality is associated with greater cortical thinning during early-to-middle adolescence, followed by attenuation of this process during the transition to young adulthood^26^. Previous adolescent functional MRI (fMRI) studies revealed that prosociality is related to the temporoparietal junction^27–29^, medial temporal lobe subsystem^27^, inferior frontal gyrus^30,31^, posterior superior temporal sulcus, temporal pole, and dorsolateral prefrontal cortex (DLPFC)^31^. A recent meta-analytic fMRI study showed that cortical regions including middle cingulate cortex, DLPFC, dorsal posterior cingulate cortex, and ventromedial prefrontal cortex are consistently engaged by PB^32^. In addition, of note, previous MRI studies for adults have revealed positive associations of amygdala volume with the size and complexity of social networks^33^ and cooperation^34^, and the amygdala is recognized as a hub in the brain networks supporting social life^35^. We thus hypothesize that the adolescent amygdala is involved in prosocial development^36^. The effect of birth order on neuroanatomical and neurofunctional development during adolescence is also still unclear. Among the above-mentioned brain regions relevant to PB, the amygdala is relatively unlikely to be volumetrically altered during early adolescence by genetic factors compared to the other cortical regions^37,38^, which implies a hypothesis that the amygdala may be more likely to be influenced by environmental factors such as birth order. Given that birth order affects sibling competition, which may cause stress^5^, as well as responses to stress^39^, and that the amygdala is involved in responses to stressful environments^40^, birth order may influence the maturation of the amygdala. Furthermore, sex differences in prosociality are present during adolescence, indicating that girls are more prosocial than boys^22,23^. Moreover, sex may moderate the association of amygdala volume^41^ and functional connectivity (FC) with psychology in adolescence^42^. Therefore, addressing sex differences when analyzing prosociality and MRI-based amygdala measurements is crucial.

Here, we explored the effect of birth order on daily life PB (mainly towards familiar others), using a large-scale population-based birth cohort sample of adolescents [N = 3160, mean age ± standard deviation (SD) = 10.2 ± 0.3 years] recruited from the Tokyo TEEN Cohort (TTC) survey (http://ttcp.umin.jp/). PB was assessed using strengths and difficulties questionnaire (SDQ). Successively, we examined its amygdala volumetric and FC substrates [measured by sMRI and resting-state fMRI (rsfMRI), respectively], using a subsample of the TTC cohort [N = 208, mean age ± SD = 11.6 ± 0.7 years (sMRI); N = 183, mean age ± SD = 11.7 ± 0.7 years (rsfMRI)]. This type of research strategy is termed "population neuroscience"^43^ and minimizes selection bias derived from laboratory-based neuroimaging studies, which represents the strength of our study.

## Results

### Study I: Population-based birth cohort study

**Figure 1:**
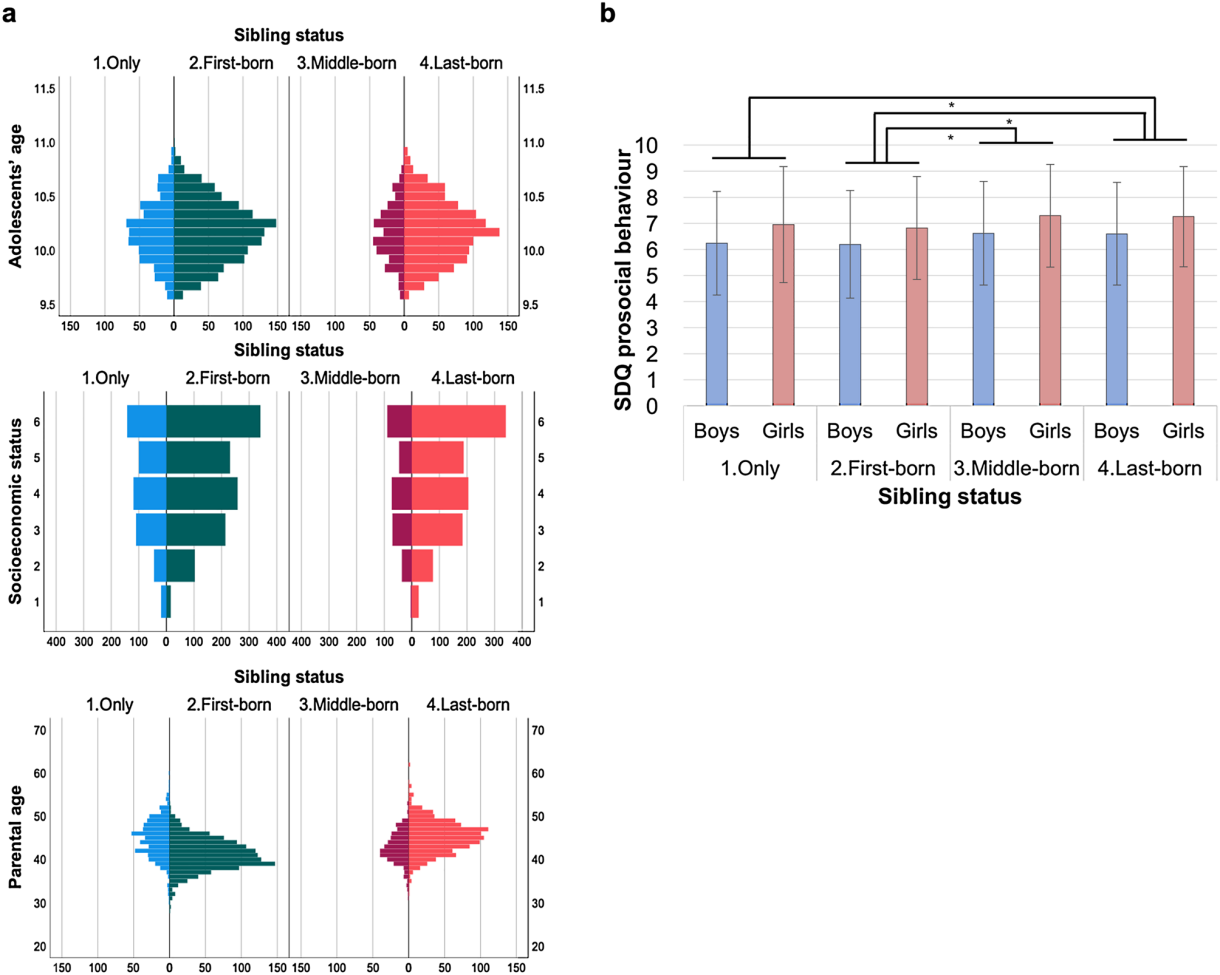
The association between sibling status and prosocial behavior (Study I). (**a**) Histograms of adolescents’ age, socioeconomic status, and parental age in each sibling status group are shown. (**b**) The means and standard deviations of the Strengths and Difficulties Questionnaire (SDQ) prosocial behavior score in each sibling status and sex group are illustrated (*: Bonferroni-corrected *p* < 0.05).

First, we investigated the effect of birth order on PB, using a large-scale population-based birth cohort sample of adolescents (N = 3160) recruited from the TTC survey^44^. We created a new birth order variable called “sibling status,” which was classified as only, first-born, middle-born, or last-born. The demographic details are described in Fig. 1a and Supplementary Table S1. In a preliminary analysis, an absence of sex difference in sibling status was found (*p* = 0.77). Furthermore, significant effects were observed for the sibling status on the SES (*p* = 7.5 × 10^−3^), and parental age (*p* = 1.5 × 10^−133^), but not on adolescents’ age (*p* = 0.74).

The effects of both sibling status and sex as well as their interaction term on SDQ PB score were investigated, controlling for adolescents’ age (Model 1, main model, N = 3156), additionally for SES (Model 2, N = 3032) and additionally for parental age (Model 3, N = 2841). Significant main effects of sibling status (Models 1–3: *p* = 3.3 × 10^−6^–1.4 × 10^−5^) and sex (Model 1–3: *p* = 4.0 × 10^−16^–6.0 × 10^−14^) were observed, although a significant effect of the sibling status by sex interaction was not found (Models 1–3: *p* = 0.83–0.90). Specifically, girls presented a higher SDQ PB score. Post-hoc tests revealed last-born adolescents to show significantly higher SDQ PB scores than only (Models 1 and 3, Bonferroni-corrected *p* = 3.4 × 10^−2^–4.4 × 10^−2^) and first-born adolescents (Models 1–3, Bonferroni-corrected *p* = 1.6 × 10^−5^–1.2 × 10^−4^), and middle-born adolescents to show significantly higher SDQ PB scores than first-born adolescents (Models 1–3, Bonferroni-corrected *p* = 2.1 × 10^−3^–3.7 × 10^−3^). These results are summarized in Fig. 1b.

### Study IIa: Amygdala structural MRI correlates

We explored the neuroanatomical and neurofunctional correlates of prosociality in adolescence using a subset brain imaging cohort [the population-neuroscience study of the TTC (pn-TTC)]^45^. A total of 208 adolescents were enrolled in a structural MRI study. Prior to the main analysis, the existence of differences in the SDQ PB score between the participants (N = 208) and non-participants (N = 2952) of the present imaging analysis was investigated. The means and SDs were 6.8 ± 2.1 and 6.7 ± 2.0 for the participants and the non-participants, respectively, indicating an absence of significant differences in the SDQ PB score between the two groups (*p* = 0.79).

**Figure 2:**
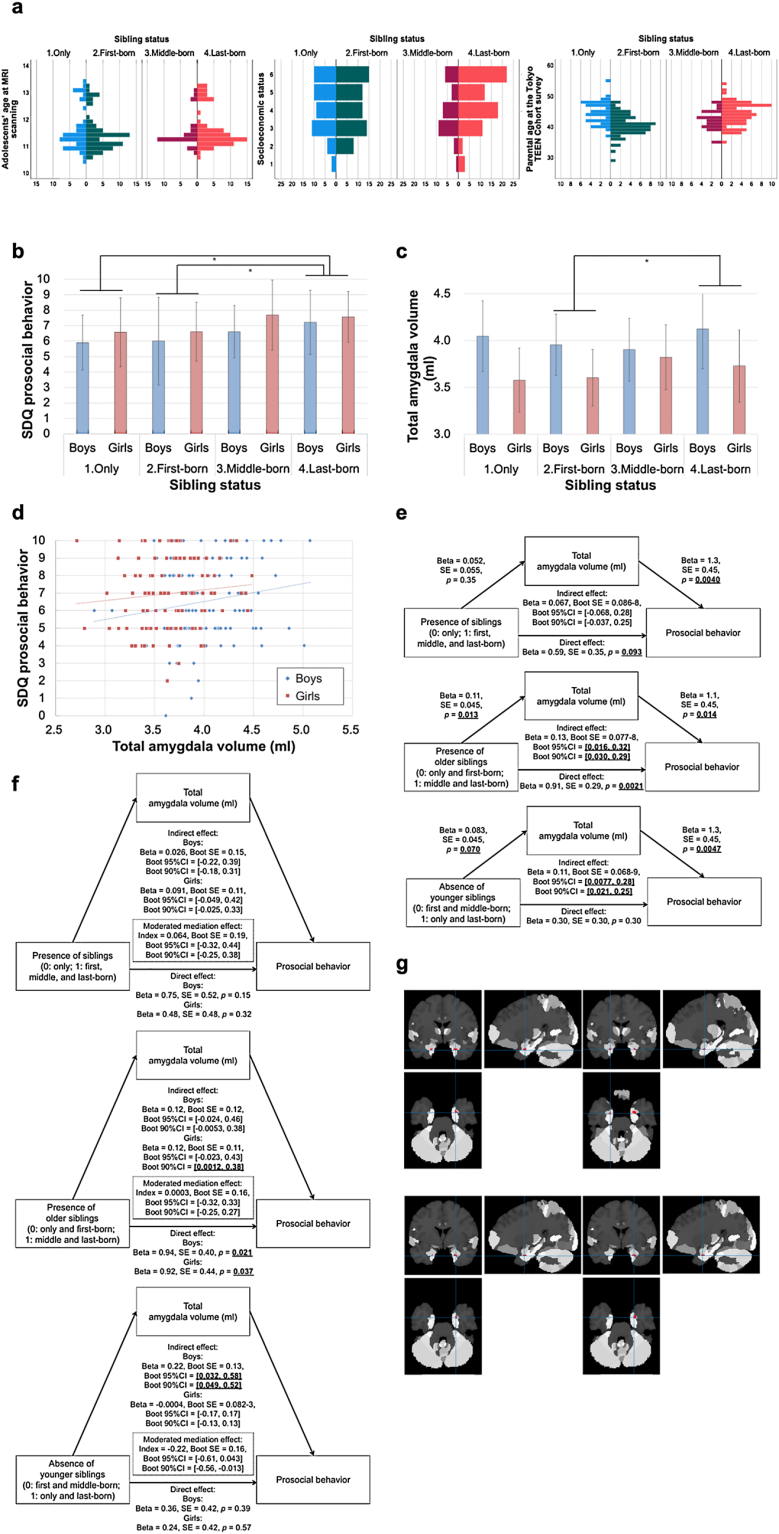
Amygdala volumetric correlates (Study IIa). (**a**) Histograms of adolescents’ age at MRI scanning, socioeconomic status, and parental age at the Tokyo TEEN Cohort (TTC) survey in each sibling status group are shown. (**b**, **c**) The means and standard deviations of the Strengths and Difficulties Questionnaire (SDQ) prosocial behavior (PB) score (**b**) and total amygdala volume (**c**) in each sibling status and sex group are illustrated (*: Bonferroni-corrected *p* < 0.05). (**d**) Total amygdala volume and the SDQ PB score are positively correlated. (**e**, **f**) The mediation effects (**e**) and sex-moderated mediation effects (**f**) of the pathways from sibling status to the SDQ PB score via the total amygdala volume are shown. (**g**) The gray matter volume in the drawn region (basolateral amygdala) is significantly positively correlated with the SDQ PB score. The analyses were adjusted for age, sex, intracranial volume (the first and second panels), additionally for socioeconomic status (the third panel), and additionally for parental age (the fourth panel).

The demographic data for Study IIa are summarized in Supplementary Table S1. Histograms of adolescents’ age at MRI scanning, SES, and parental age at the TTC survey are illustrated in Fig. 2a. The effects of both sibling status and sex as well as their interaction term on SDQ PB score were investigated, controlling for adolescents’ age (Model 1, main model), additionally for SES (Model 2, data from 5 participants were missing), and additionally for parental age (Model 3, data from 19 participants were missing). Significant main effects of the sibling status (Model 1: *p* = 5.0 × 10^−3^, Model 2: *p* = 9.0 × 10^−3^, Model 3: *p* = 1.5 × 10^−2^) and sex (Model 1: *p* = 3.5 × 10^−2^, Model 2: *p* = 3.4 × 10^−2^, Model 3: *p* = 2.9 × 10^−2^) were observed; a significant effect of the sibling status by sex interaction was not found instead (Model 1: *p* = 0.88, Model 2: *p* = 0.87, Model 3: *p* = 0.94). Specifically, girls presented higher SDQ PB scores. Post-hoc multiple comparison tests revealed last-born adolescents to show significantly higher SDQ PB scores than only (Models 1–2, Bonferroni–corrected *p* = 1.2 × 10^−2^–2.1 × 10^−2^) and first-born adolescents (Models 1 and 3, Bonferroni–corrected *p* = 1.6 × 10^−2^–2.6 × 10^−2^). These results are summarized in Fig. 2b.

The effects of both sibling status and sex as well as their interaction term on the total amygdala volume were investigated, controlling for age at MRI scanning, and intracranial volume (ICV) (Model 1, main model, N = 208), additionally for SES (Model 2, N = 203), and additionally for parental age (Model 3, N = 189). Significant main effects of sibling status in Models 1 and 2 (*p* = 2.6 × 10^−2^–4.0 × 10^−2^) but not in Model 3 (*p* = 6.4 × 10^−2^) and sex in all models (*p* = 2.7 × 10^−2^–3.3 × 10^−2^) were observed, although a significant effect of the sibling status by sex interaction was not found (Models 1–3: *p* = 0.34–0.40). Specifically, boys presented a larger total amygdala volume, while post-hoc tests revealed last-born adolescents to show a significantly higher total amygdala volume than first-born adolescents (Bonferroni–corrected *p* = 4.4 × 10^−2^) in Model 1. In contrast, a significant difference in total amygdala volume between any two groups was not seen in Models 2 or 3. These results are summarized in Fig. 2c.

Furthermore, the association of the total amygdala volume with SDQ PB score was investigated, controlling for age at MRI scanning, sex, and ICV (Model 1, main model), additionally for SES (Model 2), and additionally for parental age (Model 3). A significant positive correlation between the total amygdala volume and the SDQ PB score was found (Models 1–3: *p* = 2.9 × 10^−3^–1.6 × 10^−2^). Moreover, the effect of the interaction between sex and total amygdala volume on the SDQ PB score was investigated. A significant effect was not seen in any of the models (Models 1–3: *p* = 0.67–0.95). These results are described in Fig. 2d.

Mediation analyses were performed to assess whether the total amygdala volume mediates the effect of sibling status on the SDQ PB score. Neither direct (95% confidence interval [CI] = [− 0.099, 1.3]) nor indirect effects (95% CI = [− 0.068, 0.28]) of the presence of siblings on SDQ PB score were significant. However, both direct (95% CI = [0.33, 1.5]) and indirect effects (95% CI = [0.016, 0.32]) of the presence of older siblings on the SDQ PB score were significant. In contrast, direct effects of the absence of younger siblings on the SDQ PB score were not significant (95% CI = [− 0.28, 0.89]), whereas indirect effects were significant (95% CI = [0.0077, 0.28]). These results of the mediation analysis are illustrated in Fig. 2e. In addition, the moderating effects of sex on these pathways were examined and they were not found on either the pathway from the presence of siblings (95% CI = [− 0.32, 0.44]), the presence of older siblings (95% CI = [− 0.32, 0.33]), or the absence of younger siblings to the SDQ PB score via total amygdala volume (95% CI = [− 0.61, 0.043]). These results are summarized in Fig. 2f.

In additional analyses, the amygdala subregions in which the gray matter volumes (GMVs) were significantly correlated with the SDQ PB score were explored, controlling for age at MRI scanning, sex and ICV (Model 1, main model), additionally for SES (Model 2), and additionally for parental age (Model 3), by using voxel-based morphometry. The statistical threshold was set at an uncorrected *p* < 0.001 at the voxel level and at a small-volume correction (SVC) family-wise error (FWE)-corrected *p* < 0.05 at the cluster level. In Model 1, the SDQ PB score was significantly positively correlated with the GMV in the bilateral amygdala clusters (peak voxel MNIxyz = [26 − 6 − 27] and [− 21 − 8 − 26]) (Supplementary Table S2), whose peak voxels were located in the basolateral amygdala (BLA) (Fig. 2g). In contrast, PB was significantly positively correlated with the GMV in a right amygdala cluster in both Models 2 and 3 (peak voxel MNIxyz = [26 − 4 − 27] for both models) (Supplementary Table S2), whose peak voxel was also located in the BLA (Fig. 2g).

In a contrast analysis, the effects of both sibling status and sex as well as their interaction term on the total hippocampal volume were investigated. A significant effect of neither the sibling status (Models 1–3: *p* = 0.44–0.69), sex (Models 1–3: *p* = 0.25–0.45), nor sibling status by sex interaction (Models 1–3: *p* = 0.66–0.79) was observed. These results are summarized in Supplementary Fig. S1a.

The association of the total hippocampal volume with the SDQ PB score was investigated. A significant correlation between the total hippocampal volume and the SDQ PB score was not observed (Models 1–3: *p* = 0.18–0.30). In addition, the effect of interaction between sex and total hippocampal volume on the SDQ PB score was explored. An absence of significant effects in any of the models was reported (Models 1–3: *p* = 0.58–0.69). These results are described in Supplementary Fig. S1b.

Mediation analyses were also conducted as contrast analyses to assess whether the total hippocampal volume mediates the effect of sibling status on the SDQ PB score and to examine the moderating effects of sex on these pathways. However, no significant findings were observed (data are not shown).

### Study IIb: Amygdala functional MRI correlates

A total of 183 adolescents were enrolled in a rsfMRI study. Prior to the main analysis, the existence of any differences in the SDQ PB score between the participants (N = 183) and non-participants (N = 2977) of the present imaging analysis was investigated. The means and SDs were 6.8 ± 2.2 and 6.7 ± 2.0 for the participants and the non-participants, respectively, suggesting a lack of significant differences in the SDQ PB score between the two groups (*p* = 0.49).

**Figure 3:**
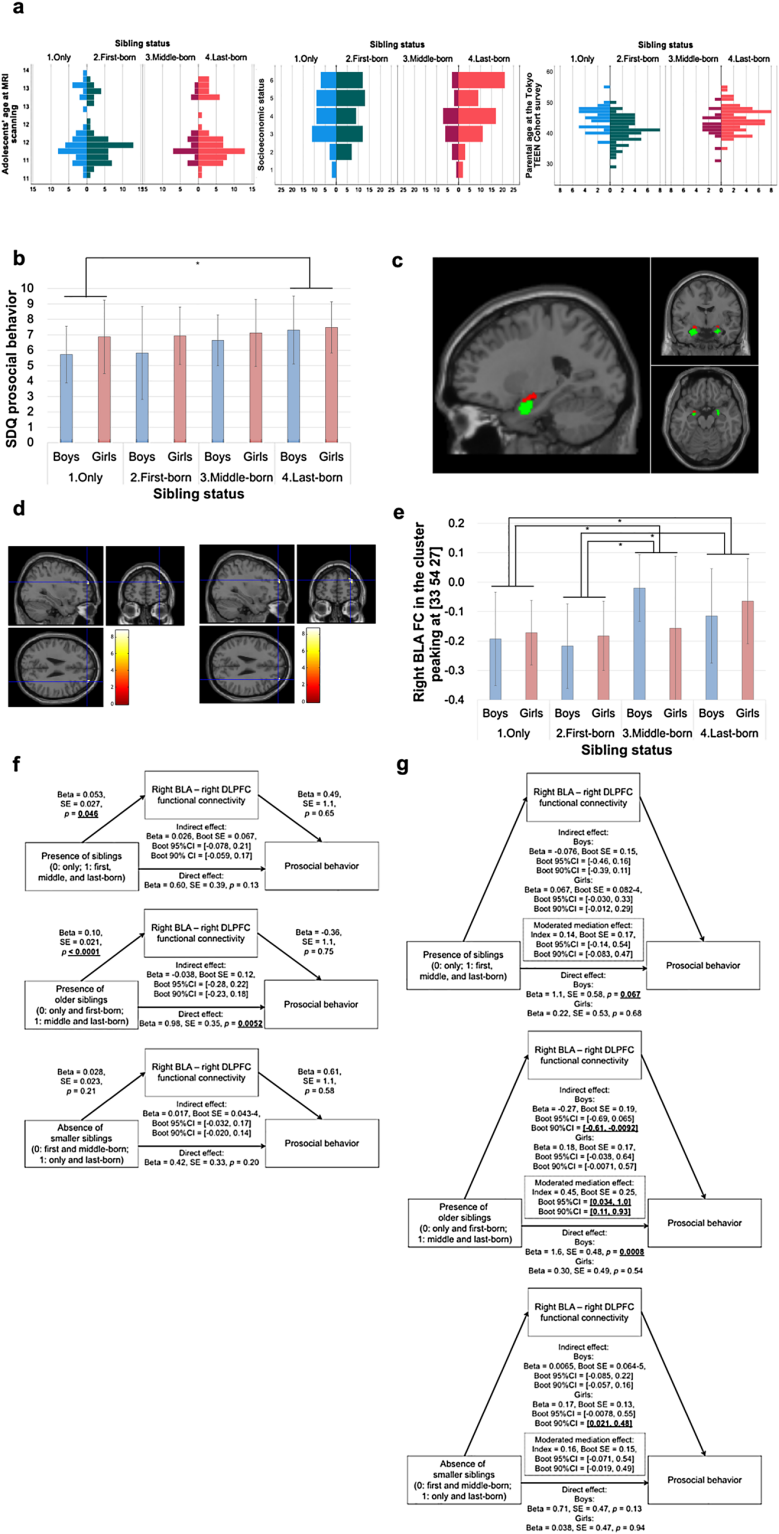
Amygdala functional correlates (Study IIb). (**a**) Histograms of adolescents’ age at MRI scanning, socioeconomic status, and parental age at the TTC survey in each sibling status group are shown. (**b**) The means and standard deviations (SDs) of the Strengths and Difficulties Questionnaire (SDQ) prosocial behavior (PB) score in each sibling status and sex group are illustrated (*: Bonferroni-corrected *p* < 0.05). (**c**) The two major amygdala nuclei, the basolateral amygdala (BLA) (green) and the centromedial amygdala (CMA) (red), were used as seed regions. (**d**) In the drawn region [right dorsolateral prefrontal cortex (DLPFC)], the sibling status has marginal and significant effects on the right BLA functional connectivity (FC), when controlling for age, sex (left), and additionally for socioeconomic status (right), respectively. (**e**) The means and SDs of the right BLA FC in the cluster described in the left panel of (**d**) are illustrated (*: Bonferroni-corrected *p* < 0.05). (**f**, **g**) Mediation effects (**f**) and sex-moderated mediation effects (**g**) of the pathways from the sibling status to the SDQ PB score via the right BLA–right DLPFC FC are shown.

The demographic data for Study IIb are summarized in Supplementary Table S1. Histograms of adolescent’s age at MRI scanning, SES, and parental age at the TTC survey are illustrated in Fig. 3a. The effects of both sibling status and sex as well as their interaction term on SDQ PB score were investigated, controlling for adolescents’ age (Model 1, main model), additionally for SES (Model 2, data from 4 participants were missing), and additionally for parental age (Model 3, data from 18 participants were missing). A significant main effect of the sibling status in Models 1 and 2 (Model 1: *p* = 3.9 × 10^−2^, Model 2: *p* = 5.0 × 10^−2^, Model 3: *p* = 7.8 × 10^−2^) and sex in all models (Model 1: *p* = 4.9 × 10^−2^, Model 2: *p* = 3.9 × 10^−2^, Model 3: *p* = 2.3 × 10^−2^) was observed; a significant effect of the sibling status by sex interaction was not found instead (Model 1: *p* = 0.64, Model 2: *p* = 0.68, Model 3: *p* = 0.79). Specifically, girls presented higher SDQ PB scores. Post-hoc multiple comparison tests revealed last-born adolescents to show significantly higher SDQ PB scores than only adolescents (Model 1, Bonferroni–corrected *p* = 5.0 × 10^−2^). These results are summarized in Fig. 3b.

Brain regions in which the sibling status had a significant main effect on the FC seeded in the four main amygdala nuclei (each side of the BLA and centromedial amygdala [CMA]) (Fig. 3c) were investigated, controlling for sex and age at MRI scanning in Model 1 (N = 183), additionally for SES in Model 2 (N = 179), and additionally for parental age in Model 3 (N = 165). The statistical threshold was set at an uncorrected *p* < 0.00025 (0.001/4) at voxel level and at an FDR-corrected *p* < 0.05 at cluster level. A significant effect of sibling status on FC between the right BLA and with the right DLPFC (peak voxel MNIxyz = [33 54 27], cluster size 13) was found in Model 2 (cluster-level FDR-corrected *p* = 3.6 × 10^−2^), whereas a trend was observed in Model 1 (cluster-level FDR-corrected *p* = 8.1 × 10^−2^) (Fig. 3d and Supplementary Table S3). No significant effects of sibling status on the right BLA FC were observed in Model 3. Post-hoc tests showed such FC to be significantly stronger in middle-born adolescents compared with only (Models 1–2: Bonferroni-corrected *p* = 4.2 × 10^−3^–1.0 × 10^−2^) and first-born ones (Models 1–2: Bonferroni-corrected *p* = 4.8 × 10^−4^–2.1 × 10^−3^) and in last-born adolescents compared with only (Model 1–2: Bonferroni-corrected *p* = 1.1 × 10^−2^–2.6 × 10^−2^) and first-born ones (Models 1–2: Bonferroni-corrected *p* = 9.2 × 10^−4^–1.5 × 10^−3^) (Fig. 3e). In contrast, a significant effect of sibling status on the FC seeded in either the left BLA, left CMA, or right CMA was not observed. Successively, brain regions in which the sibling status by sex interaction influenced the amygdala-seeded FC were examined, however, significant brain regions were not seen.

Subsequently, it was investigated whether the right BLA–right DLPFC FC was associated with the SDQ PB score using multiple regression models. No significant association was observed.

Given the finding of a region in which the sibling status had a significant effect on the right BLA FC, mediation analyses were conducted to assess whether this FC mediates the effect of the sibling status on the SDQ PB score. Neither direct (95% CI = [− 0.17, 1.4]) nor indirect effects (95% CI = [− 0.078, 0.21]) of the presence of siblings on the SDQ PB score were significant. However, direct effects of the presence of older siblings on PB were significant (95% CI = [0.29, 1.7]), while indirect effects were not significant (95% CI = [− 0.28, 0.22]). In concordance, neither direct (95% CI = [− 0.23, 1.1]) nor indirect effects (95% CI = [− 0.032, 0.17]) of the absence of younger siblings on PB were significant. These results are described in Fig. 3f. Finally, the moderating effects of sex on these pathways were investigated. Significant effects were found from the presence of older siblings (95% CI = [0.034, 1.0]), but not from either the presence of siblings (95% CI = [− 0.14, 0.54]) or the absence of younger siblings (95% CI = [− 0.071, 0.54]). In the pathway from the presence of older siblings, boys had a marginally significant negative mediation effect (90% CI = [− 0.61, − 0.0092]), while girls showed no significant mediation effect (90% CI = [− 0.0071, 0.57]). These results are summarized in Fig. 3g.

## Discussion

In the current study, we found enhanced prosociality in later-born adolescents, and observed the mediating role of larger amygdala volume and amygdala-prefrontal FC with sex-selective effects.

Later-born adolescents presented higher prosociality than earlier-born ones. This finding is line with our hypothesis and some previous results^19^, despite no significant effects of birth order on sociality in middle-aged adults^46^. We suggest that the social development in adolescence should be relatively sensitive to the birth order effect. A possible psychological mechanism of this phenomenon is that later-born adolescents may get more prosocial in order to adaptively overcome their adversity. They are relatively unlikely to feel secure due to sibling competition^7,8^ or less attachment to mothers^9^, which was also reported in chimpanzees^47^. Another possible psychological mechanism is that later-born adolescents may develop PB through interactions with older siblings^6^.

Last-born adolescents presented significantly larger amygdala volumes than first-born adolescents. Previous reports indicating that institutionalized adoptees, one extreme of the birth-order continuum, have enlarged amygdala, support our current results^48,49^. Previous studies reported that stressful events increase the volume of amygdala, especially the BLA. Decreased perceived stress is associated with decreased BLA GMVs in human adults^50^. High levels of anxiety in childhood are associated with increased amygdala, that is specifically localized to the BLA^51^. Animal studies also reported the association of stressful environment and amygdala volumetric changes. Stressor causes dendritic growth in the BLA in rats^52^. Early-weaned mice show precocious myelination in the amygdala^53^. Stress exposure increases amygdala volume as well as alters the sensitivity of hypothalamic–pituitary–adrenal (HPA) axis^54^. In sum, given that later-born children are less likely to feel secure due to sibling competition^7,8^ or diluted parental resources^4,9^, we thus suggest that a stressful or adverse family environment of later-born adolescents may increase their amygdala volumes through neuroendocrinological and microstructural neural changes.

The FC between the right BLA and the right DLPFC was significantly increased in later-born adolescents. The amygdala-DLPFC FC, a major frontolimbic pathway, is involved in emotional regulation. Although the results are mixed, a reduction of such FC is associated with not only adolescent depression^55^ but also childhood anxiety traits both in humans^56,57^ and young monkeys^57^. Later-born children are less likely to feel secure as mentioned above. Such stressful or adverse circumstances may compensatorily strengthen the emotional pathway to adapt appropriately.

Significant moderating effects of sex on the pathways from the presence of older siblings to PB via such FC were revealed. Females tend to emphasize emotion, care, and relationships, while males accentuate collective orientation and justice^58^. A recent work reported that moral elevation, a positive moral emotion triggered by witnessing other’s virtuous act, mediates the effect of moral judgment on PB to a larger extent in female young adults than in males^59^. Different types of motivation for altruism (empathy versus reciprocity) may be based on different brain networks^60^. Thus, such potential sex differences in factors related to PB may underlie the sex-moderating effects found in the current study.

Our study has some strengths. First, using a large-scale cohort dataset of more than 3000 adolescents, we successfully replicated previous results about the relationship between birth order and prosociality^19^. Second, to our knowledge, this is the first to report the association of birth order with brain regional volume and FC. Finally, we successfully revealed the neurobiological correlates of later-born adolescents’ prosociality. In contrast, our study has some limitations. First, some of the significant results were demonstrated only in one or two out of the three employed models in multiple stepwise analysis. This may be ascribed to the effect of covariates or to the difference in subject numbers across models. In fact, other factors that we did not include in this study may also possibly affect the significant relationship. Further investigations will be needed to reveal the effect of various factors on our current findings. Second, we did not reveal how stressful or adverse environments of later-born adolescents alter the amygdala volume as well as FC. Further investigations targeting stress-related factors such as psychological, physiological, and hormonal factors may overcome this limitation.

In conclusion, we found that birth order, a non-genetic environmental factor, affects adolescent social development via different neural substrates. The current study provides novel insights into the developmental neuroscience of birth order, enriching the long history of the research field on birth order psychology.

## Methods

All methods were carried out in accordance with relevant guidelines and regulations.

### Ethics

A written informed consent was obtained from each participant and their primary carer before participation. All the protocols were approved by the research ethics committees of the University of Tokyo, Faculty of Medicine (Approval Nos. 3150, 10057, and 10069), Tokyo Metropolitan Institute of Medical Science (Approval No. 12–35), and the Graduate University for Advanced Studies (SOKENDAI) (Approval No. 2012002).

### Study I: Population-based birth cohort study

The data were drawn from the TTC study, that was launched in 2012. It is a large-scale longitudinal general population-based survey to elucidate adolescent development^44^. The first wave of the TTC survey targeted a sample of youths of 10 years of age and assessed a broad range of factors, including biological, psychological, and social factors, allowing for investigations on youth’s mental and physical health development. The participants were recruited in three municipal areas of Tokyo, by using the resident register. Of the 18,830 adolescents born between September 1, 2002 and August 31, 2004, 14,553 pairs of adolescents and parents were randomly selected to participate in the survey. However, 4319 pairs could not be contacted and 5756 pairs refused to participate, restricting our population sample to 4478 pairs and revealing a response rate of 43.8%. Thereafter, this group was entered in the Tokyo Early Adolescence Survey (T-EAS), which is a cross-sectional survey. Details on the recruitment and sampling procedures of the T-EAS are provided elsewhere^44^. Among the 4478 pairs of participants in the T-EAS survey, 3171 agreed to participate in the longitudinal study. Therefore, a total of 3171 pairs were included in the TTC survey.

The Japanese version of the parent-reported SDQ, a widely used screening instrument for mental health state among children aged 4–16 years old, was used to assess adolescents’ PB^61^. In the SDQ, PB is associated with an adolescent's ability to relate well with peers, favoring actions which mainly benefit familiar others^62^. The SDQ PB subscale includes five items: ‘considerate of other people's feelings’, ‘shares readily with other children’, ‘helpful if someone is hurt, upset or feeling ill’, ‘kind to younger children’, and ‘volunteers to help others.’ Its total score ranges from 0 to 10 (low to high prosociality).

To date, most studies have analyzed the effects of birth order and sibling number separately. The present study cross-classified birth order (1st–6th borns) with sibling number (0–6 siblings), capturing all the combinations of sibling status. This new variable (hereafter labelled as sibling status) was classified into the following: 559 (17.6%) only adolescents (adolescents without siblings), 1214 (38.3%) first-born adolescents (adolescents with only younger siblings), 332 (10.5%) middle-born adolescents (adolescents with both older and younger siblings), and 1066 (33.6%) last-born adolescents (adolescents with only older siblings).

An ecological theory posits that child development should be viewed in the milieus of ecological systems, meaning that both child’s characteristics and environmental variables influence his/her development. The present analysis adjusted for the potential confounding effects of adolescents’ age, sex, family SES, and parental age. The indications from previous reports on the potential age and sex differences in prosocial development were considered. Specifically, the SES was hypothesized to have both direct and indirect influences on prosocial development^63^, also considering the traditional argument stating that parents with a lower SES tend to have an increased number of children. In the present analysis, the SES was classified based on the annual household income with the following ordinal variables: (1) ≤ Japanese Yen (JPY) 1,999,999, (2) JPY 2,000,000–JPY 3,999,999, (3) JPY 4,000,000–JPY 5,999,999, (4) JPY 6,000,000–JPY 7,999,999, (5) JPY 8,000,000–JPY 9,999,999, and (6) ≥ JPY 10,000,000 [JPY 100 ≈ United States Dollar (USD) 1]. Additionally, both the mother’s and father’s ages were included as confounding factors, given that parental age can alter the prosocial development in the offspring and differ among the sibling status group due to a similar age (i.e., the birth order is high, the parental age is high). To prevent collinearity issues, parental age was defined as the mean of the mother’s and father’s ages and was included in the statistical models of the current study.

The SDQ PB score data were missing for 11 adolescents, and thus 3160 adolescents were enrolled in Study I. The demographic data for Study I are summarized in Supplementary Table S1. Histograms of adolescents’ age, SES, and parental age are illustrated in Fig. 1a.

The significance level was set at *p* < 0.05 (two-tailed). Statistical analyses were conducted using SPSS Statistics (IBM, New York, USA). In the preliminary analysis, chi-square tests were performed to assess whether four groups of sibling status differed depending on sex. Furthermore, one-way non-parametric Kruskal–Wallis ANOVA tests were conducted to assess whether the effects of sibling status on adolescents’ age, SES, and parental age were significant. With regard to the main analysis, two-way non-parametric Quade's rank ANCOVA tests were performed^64^, with both the sibling status and sex as well as their interaction term set as explanatory variables and the SDQ PB score as a dependent variable, controlling for adolescents’ age (Model 1, data from 4 participants were missing). In addition, the SES (Model 2, data for 128 participants were missing) and parental age (Model 3, data for 319 participants were missing) were incrementally included as nuisance covariates to assess whether they could alter the effect of sibling status. Such multiple stepwise analysis could achieve multifaceted results by assessing unique effects at each step. Finally, post-hoc tests with Bonferroni correction were conducted to correct for multiple comparison tests and determine significant differences between any two groups.

### Study IIa: Amygdala structural MRI correlates

Participants of the pn-TTC study were subsampled from a larger participant group of the TTC study^45^. The latter is a large-scale longitudinal population-based cohort survey conducted in the Tokyo metropolitan area, in which 3171 early adolescents participated. Those who showed interest in the pn-TTC study were regarded as candidate participants, and some subjects were enrolled in the pn-TTC study approximately one year following their participation in the TTC study. The target sample size of the pn-TTC study was set to approximately 300. To check for any non-negligible sampling bias in the pn-TTC subsample, the basic attributes acquired in the TTC study were compared between the participants and the non-participants in the pn-TTC study and significant differences in adolescents’ age, sex, SES, and intelligence quotient were not found (*p* > 0.20). Thus, the pn-TTC subsample was confirmed to be demographically and socioeconomically representative of the original TTC study population. During their first visit, participants were introduced to a mock scanner to acclimatize to the MRI scanner environment and practice lying still during the scan. Thereafter, participants underwent MRI scanning in their second visit. The exclusion criteria for participation included: (a) evident psychiatric or neurological disorders [e.g., ASD, attention-deficit hyperactivity disorder (ADHD), Down syndrome, or epilepsy]; (b) visual or auditory impairments (except for myopia); (c) endocrinological diseases, or diseases that might have an effect on the HPA axis function (e.g., diabetes mellitus, thyroid disease, or renal dysfunction), gonadotropic dysfunction, or adrenal dysfunction; (d) recent or long-term use of drugs that might influence the central nervous system (e.g., steroid hormones and antihistamines); (e) history of head trauma with loss of consciousness for five minutes or more; (f) metal implants in the body (except titanium); and (g) unrest during the MRI practice session in the first visit.

MRI scanning was performed on a Philips Achieva 3 T system (Philips Medical Systems, Best, The Netherlands) using an eight-channel receive head coil. Each subject underwent an MRI examination, comprising fluid attenuated inversion recovery (FLAIR), rsfMRI, a T1 three-dimensional (3D) magnetization-prepared rapid gradient echo sequence (3D-MPRAGE), and MR angiography (MRA) sequences. Sagittal T1-weighted images were acquired with the following parameters; repetition time (TR) = 7.0 ms, echo time (TE) = 3.2 ms, flip angle = 9 degrees, matrix = 256 × 256, field of view (FOV) = 256 × 240 × 200 mm, voxel size = 1 × 1 × 1 mm, slice thickness = 1 mm, number of slices = 200. The total acquisition time was approximately 10 min and 42 s.

T1-weighted imaging data that had passed the quality control step were processed with Linux-based FreeSurfer software version 5.3 (http://surfer.nmr.mgh.harvard.edu)^65^. FreeSurfer provides an automated processing pipeline, through which the images of the subcortical segmentation and regional volumes of the bilateral lateral ventricles, thalamus, caudate, putamen, globus pallidus, hippocampus, amygdala, accumbens and the ICV were obtained. Briefly, this method comprises the following five steps: an affine registration with Montreal Neurological Institute (MNI) 305 space, an initial volumetric labeling, a B1 bias field correction, a high dimensional nonlinear volumetric alignment to the MNI 305 atlas, and a volumetric labeling. FreeSurfer software was used in previous studies for segmentation of adolescents’ brain images in some previous studies. However, some images could not be processed during FreeSurfer preprocessing for technical reasons.

In the quality control step, original T1-weighted images were checked by visual inspection and were excluded when reporting: (a) insufficient brain coverage (FOV problem); (b) low signal-to-noise ratios; (c) any artifacts (e.g., motion artifacts and magnetic susceptibility artifacts); and (d) any abnormal organic findings identified in either T1-weighted, FLAIR, or MRA images (e.g., large arachnoid cysts, large cavum septum pellucidum, and large arterial aneurythms). Furthermore, two independent researchers visually inspected each segmentation image after the preprocessing to exclude images with poor parcellation.

Of the 301 early adolescents who participated in the pn-TTC study, 272 [144 boys and 128 girls, mean age ± SD = 11.5 ± 0.7 years] underwent T1-weighted MRI scanning. However, 63 subjects (36 boys and 27 girls) were excluded following the identification of inadequate images in the quality control. Therefore, a total of 209 adolescents (108 boys and 101 girls) remained. Additionally, one girl was excluded due to incomplete FreeSurfer processing, whereas none of the subjects were expelled due to poor segmentation. Overall, a total of 208 adolescents (108 boys and 100 girls) were included in the final population of the present study. The demographic data for Study IIa are summarized in Supplementary Table S1. Histograms of adolescents’ age at MRI scanning, SES, and parental age at the TTC survey are illustrated in Fig. 2a. The mean interval duration between the participant in the TTC study and the MRI scanning was 16 months (ranging from 7 to 39 months).

Prior to the main analysis, the existence of any differences in the SDQ PB score between the participants (N = 208) and non-participants (N = 2952) was examined using Mann–Whitney *U*-tests. In addition, to verify the similarity of the effects of the sibling status on PB among the whole cohort (TTC) and the present analysis sample, the same analysis conducted in Study I was performed on the participants of the present analysis (N = 208). One-way non-parametric Quade's rank ANCOVA tests^64^ with both sibling status and sex as well as their interaction term set as explanatory variables and the SDQ PB score as a dependent variable were performed, controlling for adolescents’ age (Model 1, main model). Furthermore, the SES (Model 2, data from 5 participants were missing) and parental age (Model 3, data from 19 participants were missing) were incrementally included as nuisance covariates. Finally, post-hoc tests with Bonferroni correction for multiple comparisons were conducted to determine significant differences between any two groups.

Given our a priori interest in the amygdala, the association between sibling status and amygdala volume was investigated. Hippocampus was also analyzed as a reference region. This is because the volumes of the amygdala and hippocampus are reported to be highly correlated, while they have different specific functions^33,66^. Two-way non-parametric Quade's rank ANCOVA tests, with the sibling status and sex as well as their interaction term set as explanatory variables and the total amygdala (as well as hippocampal) volume as a dependent variable, were performed. Age at MRI scanning, and ICV were included in this model as nuisance variables (Model 1, main model). Furthermore, the SES (Model 2, data from 5 participants were missing) and parental age at the TTC survey (Model 3, data from 19 participants were missing) were incrementally included as nuisance covariates. Finally, post-hoc tests with Bonferroni comparison for multiple comparisons were conducted to determine significant differences between any two groups.

We also investigated whether a reciprocal association between the amygdala (and, as a reference region, hippocampal) volume and the SDQ PB score existed. Linear regression analyses, with the total amygdala (as well as hippocampal) volume set as an explanatory variable and the SDQ PB score as a dependent variable, were performed. Sex, age at MRI scanning, and ICV were included in this general linear model as nuisance variables (Model 1, main model). Furthermore, the SES (Model 2, data from 5 participants were missing) and parental age at the TTC survey (Model 3, data from 19 participants were missing) were incrementally included as nuisance covariates. Finally, the effect of the interaction between sex and the total amygdala (as well as hippocampal) volume on the SDQ PB score was examined.

Mediation analyses were conducted to assess whether the total amygdala (as well as hippocampal) volume mediates the effect of sibling status seen on the SDQ PB score. Since a categorical multi-level variable cannot be entered directly into a mediation regression model, we created the following three types of dummy variables regarding the sibling status and included them in the mediation models: (1) the presence of siblings (0: only; 1: first, middle, and last-born); (2) the presence of older siblings (0: only and first-born; 1: middle and last-born); (3) the absence of younger siblings (0: first and middle-born; 1: only and last-born). Sex, age at MRI scanning, and ICV were included as nuisance variables. Successively, moderated mediation analyses were performed to assess whether sex moderates these mediation effects. Age at MRI scanning and ICV were included as nuisance variables. Both mediation analyses and moderated mediation analyses were performed through the PROCESS plugin for SPSS^67^ with 5000 bootstrapped samples.

To identify the amygdala subregions associated with the SDQ PB score, a supplementary analysis was conducted on the 208 participants who were included in the main analysis. T1-weighted imaging data that had passed the quality control step were processed for voxel-based morphometry analysis using the Statistical Parametric Mapping (SPM) 12 software (http://www.fil.ion.ucl.ac.uk/spm/)^68^ running on MATLAB 2014b (MathWorks, Natick, MA) on Mac Operating System (OS). Initially, the image origin was set to the anterior commissure. Subsequently, the MRI images were segmented using the default segmentation algorithm “new segment” in SPM12. In the segmentation module, the ‘light regularization’ was selected for the bias regularization item. Thereafter, the diffeomorphic anatomical registration through exponentiated lie (DARTEL) normalization toolbox was employed to spatially normalize the gray matter (GM) segmentation maps to the MNI space, with the Jacobian determinant modulation applied to preserve the absolute GMV. Finally, the normalized GM maps were smoothed with an 8-mm full width at half maximum (FWHM) Gaussian kernel. The total GM, white matter (WM), and cerebrospinal fluid (CSF) volumes were calculated from each modulated normalized image by using the “get_totals.m” function (http://www0.cs.ucl.ac.uk/staff/g.ridgway/vbm/get_totals.m). Successively, the ICV was obtained by summing up the GM, WM and CSF volumes. The brain regions in which GMVs were significantly correlated with the SDQ PB score were explored. Specifically, the voxel-based multiple regression was used to investigate the effect of the SDQ PB score on smoothed modulated normalized GM images, with age at MRI scanning, sex and ICV (global normalization) included as nuisance regressors (Model 1, main model). Furthermore, the SES (Model 2, data from 5 participants were missing) and parental age (Model 3, data from 19 participants were missing) were incrementally included as nuisance covariates. Given our a priori interest in the amygdala, an SVC was applied to this region using an anatomical region of interest (ROI) binary mask for the bilateral amygdala. This was created as a summation of four amygdala subregions (i.e., superficial, basolateral, centromedial, and amygdalostriatal transition area)^69^ with SPM Anatomy toolbox (http://www.fz-juelich.de/inb/inb-3/spm_anatomy_toolbox), which provides cytoarchitectonic anatomical probability maps of the amygdala subregions. The statistical threshold was set at an uncorrected *p* < 0.001 at the voxel level and at an SVC FWE-corrected *p* < 0.05 at the cluster level. Finally, the locations of significant peaks were determined with the SPM Anatomy toolbox, through the calculation of the maximum probability map^70^.

### Study IIb: Amygdala functional MRI correlates

The participant recruitment for Study IIb followed the same procedure as Study IIa.

All rsfMRI data were acquired with a gradient-echo echo-planar imaging (EPI) sequence with the following parameters: TR/TE, 2500 ms/30 ms; flip angle, 80°; acquisition matrix, 64 × 59; FOV, 212 × 199 × 159 mm; voxel size, 3.31 × 3.37 × 3.20 mm; slice thickness, 3.20 mm; and slice gap, 0.8 mm. Each brain volume consisted of 40 axial slices, and each functional run contained 250 image volumes preceded by four dummy volumes, resulting in a total scanning duration of 10 min and 40 s. Participants were asked to stay awake, not to focus their thoughts on anything specific (as far as possible), and to keep their eyes on a fixation point at the center of the screen during the rsfMRI scanning.

All rsfMRI data preprocessing was performed with data processing assistant for resting-state fMRI (DPARSF) software version 4.4^71^, which is a MATLAB toolbox, using Mac OS. The first 10 frames were removed to allow signal stabilization. Functional imaging data were corrected for slice timing and spatially realigned to the middle slice. Subsequently, images were then normalized to the MNI space, through parameters derived from the normalization of the coregistered T1-weighted images computed by New Segment and DARTEL in SPM12 (resulting in an isotropic voxel size of 3 mm^3^), smoothed with a FWHM kernel of 4 mm, and bandpass filtered (0.01–0.1 Hz). The following nuisance signals were regressed out of the time course of each voxel: (a) head motion time series estimated with the Friston 24-parameter model (head motion parameters from realigned data including 6 head motion parameters, 6 head motion parameters one time point before, and the 12 corresponding squared items); (b) head motion scrubbing regressors (time points with more than 0.5 mm of framewise displacement and 1 back and 2 forward neighboring points were modeled as a regressor); and (c) white matter, cerebrospinal fluid, and global signals. Finally, time course data were processed for each of the both sides of the two major amygdala nuclei, namely the BLA and the CMA. Prior to this processing, ROI images for each of these amygdala subregions were created using SPM Anatomy Toolbox with cytoarchitectonically defined probability maps^69^, only including voxels with a 50% or higher probability of belonging to each subregion (Fig. 3c), which were resampled to an isotropic voxel size of 3 mm^3^. Intra-regional correlation coefficients were calculated between every region-voxel pair and connectivity maps were created. The correlation coefficients representing FC were converted to z-scores with Fisher’s transformation. Finally, participants’ head motion parameters during the rsfMRI scanning were recorded.

Subjects were excluded when presenting the following: (a) any abnormal organic findings identified in either T1-weighted, FLAIR, or MRA images (e.g., large arachnoid cysts, large cavum septum pellucidum, and large arterial aneurythms); (b) rsfMRI data with a maximum translation excess of 3 mm or a maximum rotation excess of 3 degrees in any direction; and (c) connectivity maps with outlying values.

Of the 301 early adolescents who participated in the pn-TTC study, 257 (136 boys and 121 girls, mean age ± SD = 11.6 ± 0.7 years) underwent rsfMRI scanning. However, one girl who did not complete the T1-weighted imaging scanning was excluded. Furthermore, 26 subjects (15 boys and 11 girls) with abnormal organic findings, 43 subjects (26 boys and 17 girls) presenting large head motion, and 4 subjects (2 boys and 2 girls) with outlying values in their connectivity maps were also excluded. Overall, 183 early adolescents (93 boys and 90 girls, mean age ± SD = 11.7 ± 0.7 years) were analyzed in the present study. The demographic data for Study IIb are summarized in Supplementary Table S1. Histograms of adolescents’ age at MRI scanning, SES, and parental age at the TTC survey are illustrated in Fig. 3a. The mean interval duration between the participant in the TTC study and the MRI scanning was 17 months (ranging from 7 to 39 months).

Prior to the main analysis, the existence of any differences in the SDQ PB score between the participants (N = 183) and non-participants (N = 2977) was examined using Mann–Whitney *U*-tests. In addition, to verify the similarity of the effects of the sibling status on PB among the whole cohort (TTC) and the present analysis sample, the same analysis conducted in Study I was performed on the participants of the present analysis (N = 183). One-way non-parametric Quade's rank ANCOVA tests with sibling status and sex as well as their interaction term set as explanatory variables and the SDQ PB score as a dependent variable were performed, controlling for adolescents’ age (Model 1, main model). Furthermore, the SES (Model 2, data from 4 participants were missing) and parental age (Model 3, data from 18 participants were missing) were incrementally included as nuisance covariates. Finally, post-hoc tests with Bonferroni correction for multiple comparisons were conducted to determine significant differences between any two groups.

In the second-level whole-brain analysis of rsfMRI data, the statistical threshold was set at an uncorrected *p* < 0.001 at voxel level and at a false discovery rate (FDR)-corrected *p* < 0.05 at cluster level. Further, in the second-level ROI analysis of rsfMRI data using a SVC, the statistical threshold was set at an uncorrected *p* < 0.001 at voxel level and at an FWE-corrected *p* < 0.05 at cluster level, given that the FWE correction better controls for false positives in such cases when compared to FDR.

The brain regions which showed a significant main effect of the sibling status on amygdala-seeded FC were examined using SPM ANCOVA analysis, with sex and age at MRI scanning included as covariates (Model 1, main model). In addition, the SES (Model 2, data from 4 participants were missing) and parental age at the TTC survey (Model 3, data from 18 participants were missing) were incrementally included as nuisance covariates. Successively, the brain regions in which the sibling status by sex interaction had an effect on amygdala-seeded FC were investigated using SPM two-way ANCOVA analysis, with age at MRI scanning included as a covariate (Model 1, main model). In addition, the SES (Model 2, data from 4 participants were missing) and parental age at the TTC survey (Model 3, data from 18 participants were missing) were incrementally included as nuisance covariates. Given the presence of four types of amygdala subregions of interest, the statistical threshold was set at an uncorrected *p* < 0.00025 (0.001/4) at voxel level and at an FDR-corrected *p* < 0.05 at cluster level. When a significant effect of sibling status was found, a weighted average of FC in the cluster was extracted and post-hoc tests with Bonferroni correction were conducted to determine significant differences in it between any two groups using SPSS software.

The brain regions that showed a correlation between the amygdala FC and the SDQ PB score were examined using multiple regression models in SPM, with sex and age at MRI scanning included as covariates (Model 1, main model). In addition, the SES (Model 2, data from 4 participants were missing) and parental age at the TTC survey (Model 3, data from 18 participants were missing) were incrementally included as nuisance covariates. Successively, the brain regions showing different regression slopes of amygdala FC on the SDQ PB score between sexes were investigated using multiple regression models in SPM, with age at MRI scanning as a covariate (Model 1, main model). In addition, the SES (Model 2, data from 4 participants were missing) and parental age at the TTC survey (Model 3, data from 18 participants were missing) were incrementally included as nuisance covariates. Given the presence of four types of amygdala subregion ROIs, the statistical threshold was set at an uncorrected *p* < 0.00025 (0.001/4) at voxel level and at an FDR-corrected *p* < 0.05 at cluster level. These analyses were also conducted using an SVC of the clusters showing a significant effect of sibling status or sibling status by sex interaction in the above analyses (if any). The statistical threshold was set at an uncorrected *p* < 0.001 at voxel level and at an FWE-corrected *p* < 0.05 at cluster level.

Mediation analyses were conducted to assess whether amygdala FC mediates the effect of the sibling status on the SDQ PB score. Sibling status was represented in the mediation models through the same three types of dummy variables of Study IIa. Furthermore, the amygdala FCs which were found to be accounted for by the effect of sibling status or sibling status by sex interaction in the above analyses were included in the mediation models (if any). Sex and age at MRI scanning were included as nuisance variables. Subsequently, moderated mediation analyses were performed to assess whether sex moderates these mediation effects. Age at MRI scanning was included as a nuisance variable. Both mediation and moderated mediation analyses were conducted using the PROCESS plugin for SPSS with 5000 bootstrapped samples.

### Power analysis

Power analyses were performed to ensure the statistically sufficient power of the current study. Medium effect sizes were selected, because a previous late adolescent study reported a moderate effect of birth order on prosociality^19^ and there is no previous report of the association between birth order and neuroimaging. Specifically, a priori power analyses (*α* = 0.05, 1 − *β* = 0.80, two-sided tests) using G*Power 3.1.9.2^72^ revealed that the medium effect sizes for a correlational analysis (*r* = 0.30) and those for a multiple regression analysis (*f*^*2*^ = 0.15)^73^ could be detected in a sample with a size of 82 and 55 subjects, respectively. These calculations ensured the adequacy of all the sample sizes in all the analyses conducted in the present study.

### Selected statistical models

We employed an appropriate statistical model for each analysis. We performed ANCOVA analysis to explore the effect of sibling status on PB as well as amygdala volume/FC. We performed regression analysis to explore the effect of amygdala volume on PB. We conducted mediation analysis to explore the mediation effect of amygdala volume/FC on the association between sibling status (dichotomized) and amygdala volume. We think that all kinds of analyses employed in this study are necessary.

## Supplementary Information

Supplementary Information 1.

Supplementary Information 2.

Supplementary Information 3.

Supplementary Information 4.

## Data Availability

The pn-TTC project is currently working on making its data available openly under the grant [Brain Mapping by Integrated Neurotechnologies for Disease Studies (Brain/MINDS) and Beyond] supported by Japan Agency for Medical Research and Development (AMED). The data supporting the findings of the current study can be requested by contacting the last author at kasaik-tky@umin.net. Applicants will be asked to fill out the data request form, which will be examined by the pn-TTC Data Resource Committee.
