# Supplementary material for: Birth order and prosociality in the early adolescent brain
Source: Sci Rep. 2021 Nov 8;11:21806. doi: 10.1038/s41598-021-01146-0 (PMC8575884; doi:10.1038/s41598-021-01146-0)
Supplement: Supplementary file 1 — Supplementary Information 1. [file 41598_2021_1146_MOESM1_ESM.pdf]

Supplementary Figure S1

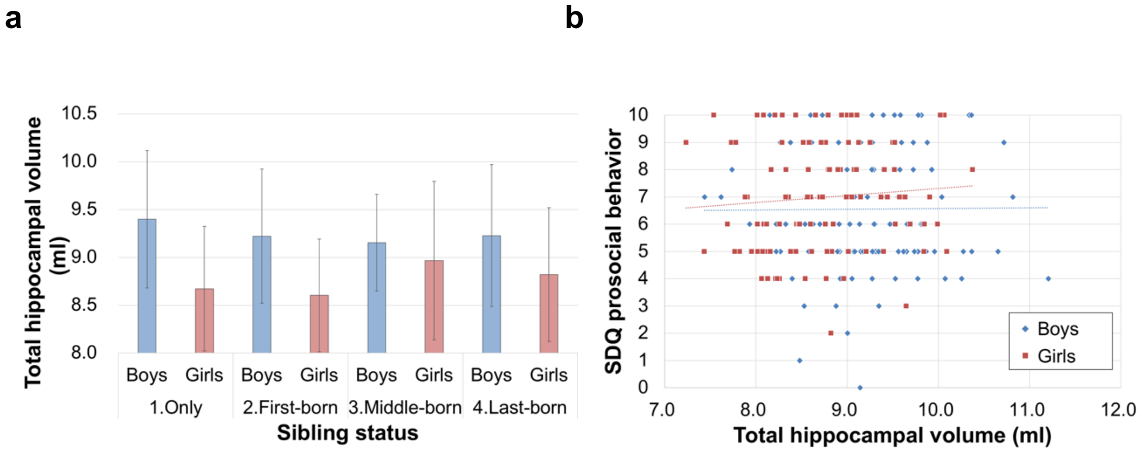

Contrast analysis. (**a**) The means and standard deviations of total hippocampal volume in each sibling status and sex group are illustrated. The hippocampal volume was calculated using FreeSurfer software version 5.3 (http://surfer.nmr.mgh.harvard.edu). Two-way non-parametric Quade's rank analysis of covariance (ANCOVA) tests were performed with the sibling status and sex as well as their interaction term set as explanatory variables and the total hippocampal volume set as a dependent variable. Models were adjusted for age and intracranial volume, additionally for socioeconomic status, and additionally for parental age. An absence of significant main effect of either sibling status, sex, or sibling status by sex interaction is observed. (**b**) The association between the total hippocampal volume and the Strengths and Difficulties Questionnaire (SDQ) prosocial behavior (PB) score is shown. Light blue dots represent boys, whereas the light red ones indicate girls. The total hippocampal volume is not significantly correlated with the SDQ PB score, adjusted for sex, age at MRI scanning, and intracranial volume, additionally for socioeconomic status, or additionally for parental age.
